# Microfiltration of Submicron-Sized and Nano-Sized Suspensions for Particle Size Determination by Dynamic Light Scattering

**DOI:** 10.3390/nano9060829

**Published:** 2019-05-31

**Authors:** Christian Ullmann, Frank Babick, Michael Stintz

**Affiliations:** Research Group Mechanical Process Engineering, Institute of Process Engineering and Environmental Technology, Technische Universität Dresden, Münchner Platz 3, D-01062 Dresden, Germany; frank.babick@tu-dresden.de (F.B.); michael.stintz@tu-dresden.de (M.S.)

**Keywords:** filtration, DLS, nanomaterial characterization

## Abstract

Dynamic light scattering (DLS) is commonly used for the determination of average particle diameters and suspension stability and popular in academics and industry. However, DLS is not considered suitable for polydisperse samples. The presence of little quantities of micrometre particles in nano and submicrometre suspensions especially affect the reliability of DLS results. Microfiltration might be a suitable method for the removal of unwanted large particles. This study investigates the effect of microfiltration on the diameter distributions as measured by DLS. Polystyrene standards (40–900 nm diameter), and monomodal silica suspensions were filtered with polytetrafluoroethylene (PTFE) membranes (0.1–1.0 µm pore size) to investigate retention properties and grade efficiency. Non-ideal materials were used to prove the results. Experiments showed that a mono-exponential decay can be achieved by filtration. A size safety factor of at least three between labeled pore size and average diameter was found to keep separation as low as possible. Filtration in order to enhance DLS for particulate submicrometre materials was considered suitable for narrowly distributed coated titania and kaolin powder. In a regulatory context, this might have an impact on considering a substance false positive or false negative according to the European Commission (EC) recommendation of a definition of the term nanomaterial.

## 1. Introduction

Dynamic light scattering techniques (DLS) have been used for many years in nanoparticle research. The determination of particle diameters and particle size distributions (PSD) by DLS is quite fast, and this technique is applicable to most colloidal dispersions [1]. The uncertainty of the particle diameter was 5% for colloidal silica IRMM-304 in a full validation study [2]. Therefore, DLS has been used for inter-laboratory studies and the development of further certified reference materials [3,4,5,6].

DLS measures the intensity fluctuation of light scattered from a sample of diffusing particles. The frequency or relaxation time of this fluctuation correlates with the particles’ diffusion coefficient and, thus, with their size. The original measurement data are processed by established algorithms, which either compute a complete PSD (e.g., constrained regularization program CONTIN [7,8]) or its characteristic moments (e.g., method of cumulants [9]). Particle diameters determined by DLS refer to the hydrodynamic equivalent diameter, and PSDs are intrinsically intensity-weighted, even though a conversion to number- or volume-weighted distributions can be carried out. DLS instruments usually use Mie’s scattering theory [10] for conversion. The simple application of Mie’s scattering theory is associated with assumptions. Algorithms to overcome these issues have been published—algorithms for figuring out how to calculate the hydrodynamic diameters of highly aggregated materials and how to determine the size distribution of microgel suspensions, for example [11,12,13]. 

DLS instruments are not considered suitable for particle diameter and PSD determinations of highly disperse samples [14,15,16,17]. Contamination leads to a broader PSD and is one of the main reasons for uncertainties of particle size measurements in general [18]. In particular, by releasing high inputs of energy into small suspension volumes by means of ultrasonic probe sonication yields the risk of contamination by the abrasion of the ultrasonic probe and sets practical limits [19,20,21,22]. This debris comprises nanoparticles as well as coarse particles in the micrometre size range [23,24,25]. Contamination has a significant impact on the sensitivity to small particles [26]. Micrometre particles significantly affect the correlation function of delay time. Studies have shown that DLS was not able to detect acrylic nanoparticles (70 nm in diameter) if there were 1% by number particles in the sub-micrometre range (390 nm) [27]. With regard to the current state of the art, DLS results always need to be interpreted carefully, e.g., by comparing them to electron microscopy (EM) images [16] or results obtained by field flow fractionation (FFF) techniques [28]. 

An approach that allows one to get access to the smallest particle fractions by DLS is to apply microfiltration to suspensions before measurement [29]. In theory, the removal of particles in the micrometre or upper submicrometre size range might improve the correlation function but should not significantly influence number-weighted related statistic values of submicrometre or nanosuspensions, e.g., the median diameter x_50,0._ Conversion issues are comprehensively discussed in the literature [30,31,32]. Microfiltration for the analysis of suspensions has already been used, e.g., for quantifying engineered nanoparticles in the aquatic environment [33,34,35], as well as for nanoparticle analysis in food and cosmetics [28,29]. To establish DLS in the regulatory context, filtration studies were conducted to test the risk of providing a false-positive classification of a material as a nanomaterial [36]. International Organization for Standardization (ISO) standard ISO 14887:2010 describes procedures for dispersing powders in liquids and is applicable in the particle size range between 0.05 and 100 µm [37]. The use of DLS instruments is standardized in ISO 22412:2008 [38] and American Society for Testing and Material (ASTM) standard ASTM E2490-09 [39]. ISO 22412:2008 only mentions the possibility of sample filtration in chapter B.5. There are concerns that a large quantity with considerably smaller diameters than the nominal pore size value might be removed too, e.g., by deep-bed filtration.

Theoretically, microporous filters should retain nearly all particles greater than the labelled value. Membrane filters are routinely used to remove particulate contamination from solutions, e.g., prior to chromatographic analysis [40]. Usually, filters are labelled with a nominal or an absolute pore size value, which is probably misused to a great extent in public. Most membrane pores have unequal and statistically distributed pore sizes. The nominal value describes the ability of the filter to retain the majority of the particles at this pore size and larger. The absolute pore size denotes the size of the smallest particle completely retained. The retention properties are mainly affected by the filter properties (e.g., porosity and its filter area), as well as by the suspensions physicochemical characteristics (e.g., viscosity, pH, and ionic strength) and the process conditions (e.g., differential pressure and temperature) [41]. Many types of materials can be used, and there are various recovery rates for different filter materials, but polytetrafluoroethylene (PTFE) shows the best resistance against strong acids and other aggressive chemicals [42]. 

This study investigates the performance of preparative filtration of nano- and submicrometre suspensions for the purpose of PSD analysis by means of DLS. The objectives of preparative filtration are to increase the sensitivity of nanoparticle detection and to improve the accuracy of quantitation to obtain reliable PSDs by means of DLS. The grade efficiencies of membranes are determined by studies with ideal monodisperse suspensions in the size range 40–900 nm diameter. The effects of various solid contents in retention are evaluated with colloidal silica suspensions. The results re-assessed by filtration with non-ideal substances. Experimental data of blank samples and some non-ideal materials (BaSO_4_ and kaolin) were published in a public technical report [36]. The filtration experiments on coated titania were repeated for this publication. In this article, these results are comprehensively discussed regarding the determined grade efficiencies, filtration predictability, membrane characteristics, and new filtration results.

## 2. Materials and Methods 

### 2.1. Materials

#### 2.1.1. Reference Materials

Nanosphere^®^ size standards by Duke Scientific were used as reference materials (RM). These suspensions contain pre-dispersed spherical polystyrene particles with an original solids content of 1 wt%. The certified particle diameters were in the size range between 40 and 900 nm.

#### 2.1.2. Representative Test Materials

Representative test materials (RTMs) are materials that passed studies on homogeneity and stability and can be used as benchmarks for examples of new or modified size determination methods [43]. A monodisperse silica suspension (Levasil^®^ 50/50%, H.C. Starck GmbH) was used for investigations on the retention properties, depending on the solid content of the suspension. Levasil^®^’s cumulants diameter is approximately 104 nm. The other RTMs were powders of polydisperse substances provided by JRC-IRMM. An overview on the materials is given in Table 1

#### 2.1.3. Membranes

Microporous hydrophilic OMNIPORE^®^ membranes (diameter 47 mm) by Merck KGaA were used in a dead-end filtration process. OMNIPORE^®^ membranes are PTFE disc filters and are available in a suitable pore size range (0.1–10 µm). PTFE filters are expected to be compatible for a wide range aquatic and organic solvents, as well as alkaline and acid solutions, and are considered as biologically and chemically inert. No chemical interaction was expected with any solvent used in this study. The manufacturers filter codes and the labelled pore sizes of membranes investigated in this study were JAWP (1.0 µm), JHWP (0.45 µm), JGWP (0.20 µm), and JVWP (0.10 µm). According to documentation, the filter porosity was 80% for all membranes. The membrane thickness varied and depended on the denoted pore size (labelled size); the thicknesses were, respectively, 85 µm (1.0 µm), 65 µm (0.45 µm and 0.20 µm), and 30 µm (0.10 µm).

### 2.2. Desagglomeration

The deagglomeration (disassembling agglomerates) in the RTM suspensions was conducted by means of ultrasonic dispersion with the UDS 751 device (Topas GmbH). The effective dispersion of original dry nano-powders into a liquid requires a high intensity method to break up assemblies of particles and to ensure the absence of agglomerates. A probe (7 mm diameter) was used at high intensity (80% amplitude) to treat the suspension batch (20 mL volume). Meanwhile, an ice bath cooled the suspension.

### 2.3. Membrane Characterization

#### 2.3.1. SEM Images

Pieces of PTFE OMNIPORE^®^ membranes were cut (3 × 3 mm) and sputtered with platinum in an argon atmosphere (45 seconds, 20 mA, 300 V). The expected coating was 1–2 nm. The used instrument for images is a Gemini 982 SEM by Carl Zeiss AG (Jena, Germany). The voltage was 4.0 kV.

#### 2.3.2. Pore Size Analysis

The pore size meter PSM 165 by TOPAS GmbH was used to determine the pressure drop across the membrane as a function of the gas flow rate through the membrane. The measurements were conducted for the dry and the wetted membrane. The test procedures are described in the American standards ASTM E 1294-89 [44] and ASTM F 316-03 [45]. The bubble point indicates the opening of the largest liquid-filled pore, and the wet membrane will become gas permeable at this certain gas pressure. By increasing the gas flow rate, it is possible to determine the pore size distribution. The physical model is described in the literature [46]. Topor (Topas GmbH, surface tension 16.0 mN·m^−1^, density 1.9 g·mL^−1^, both at 25 °C) was used as wetting fluid. The adapter sizes were of 11 mm and 6 mm diameter. Preliminary measurements showed that an appropriate pinhole aperture is needed to avoid any curvature of the membrane. The bubble point was determined manually. According to the vendor’s documentation, the measuring range is 0.3–130 µm. However, measurements were carried out all four membranes. Membranes were cut (approximately 40 × 40 mm), and the pieces were fixed in the instrument. The software was PSMwin version 4.2 by TOPAS GmbH. 

### 2.4. Sample Preparation Procedures

#### 2.4.1. Blank Samples 

In this study, blank samples were simple aqueous solutions with surfactants but without dispersed particulate materials. Glass beakers (50 ml) were filled with a tetra sodium pyrophosphate solution (TSPP, 120 µl, 50 g·kg^−1^) and deionized water (20 mL, 18.3 MΩ·cm^−1^). The blank solution was sonicated by means of probe sonication (12 min) as described in Section 2.2 in order to reproduce the deagglomeration conditions of RTMs. Three independently prepared replicates of each material were measured in this study.

#### 2.4.2. Procedures for Reference Materials

Nanosphere^®^ size standards (200 µL) were diluted with a sodium chloride (AnalaR Normapur, VWR Prolabo^®^) solution (19.8 mL, 0.01 M) to a final solid content of 100 ppm. This initial concentration is suitable to be low enough to avoid multiple scattering during the DLS measurement and high enough to prove a possible separation during the filtration. The standards were gently sonicated in an ultrasonic bath (1 min) in their original bottle in order to homogenize the sample. After dilution, the samples were gently sonicated in an ultrasonic bath (1 min) again for the same reason.

#### 2.4.3. Monomodal Material

Four Levasil^®^ silica suspensions with solid contents between 350 and 3500 ppm and pH 9.0 were prepared. These initial concentrations are low enough to avoid multiple scattering during the DLS measurement because of the low refractive index of silica [47]. The necessary amount of original silica suspension was put into a glass beaker (50 mL), and a KOH solution (pH 9.0) was added. The suspension’s pH was checked after a few minutes, and a subsequent adjustment with a KOH solution (pH 10) was conducted in order to reach pH 9, which is required to consider the silica suspension stable. Afterwards, the samples were gently sonicated in an ultrasonic bath (1 min) in order to homogenize the sample. 

#### 2.4.4. Polydisperse Materials

The suspension of the RTMs BaSO_4_ fine and ultrafine, coated titanium dioxide, and kaolin samples were prepared according to the sample preparation procedures published by Gilliland et al. 2016 [48]. The literature indicates that the suspensions are considered stable for at least 30 min. This timescale is sufficient for the accomplishment of the required preparation and measurement tasks (filtration, sample taking, temperature equilibration of the sample, and DLS measurement).

In the case of BaSO_4_ and coated titanium dioxide, the required mass of powder was put into a glass beaker (50 mL), and a sodium hexametaphosphate solution (NaHMP, 20 mL, 2 g·L^−1^) was added to reach a solid content of 1000 ppm for the BaSO_4_ suspensions and 100 ppm for the TiO_2_ suspension. The suspensions were sonicated by means of probe sonication, as described in Section 2.2, for 11 min. In the case of kaolin, glass beakers (50 ml) were filled with kaolin powder (2 mg), a tetra sodium pyrophosphate solution (TSPP, 120 μL, 50 g·kg^−1^), and deionized water (20 mL, 18.3 MΩ·cm^−1^). Like the other RTM suspensions, the suspension was sonicated by means of probe sonication as described in Section 2.2 for 12 min. Three independently prepared replicates of each material were measured in this study.

### 2.5. Filtration Procedure

Instead of manual filtration, vacuum-assisted filtration was preferred to improve the repeatability of the filtration process. A Millipore® filtration system by Merck KGaA was used. A Büchner flask was used to collect the filtrate. The Büchner flask was connected with rubber tubing to a vacuum pump that created a partial vacuum in the flask and realized a repeatable differential pressure. The membranes were touched with tweezers only and dropped onto the porous plate (47 mm). The Büchner funnel was fixed with a spring clamp, and the suspension decanted into the Büchner funnel. The suspensions were filtered for one minute. A suspension sample (1.5 mL) was taken for DLS measurement of the recently prepared suspension before the filtration procedure, and a sample of the filtrate was taken after each filtration step. Filtrations with 0.20 µm and 0.10 µm were carried out only if the results were expected to be meaningful. 

### 2.6. DLS Measurement Procedure and Analysis

Measurements were conducted with the Malvern High Performance Particle Sizer (HPPS) (UK). The HPPS operates with a He–Ne-gas laser (vertically polarized beam, 632.8 nm) and detects scattering light in back scatting mode (173°). The intensity of the illuminating laser beam (“attenuation level” in Malvern terms) was set manually via software to conduct a filtration measurement series with the same laser beam intensity if possible. This laser beam attenuation is realized with an aperture. The measured intensities are captured as detected photons per second and are called count rate. DLS measurements were performed by DLS before and after filtration. An aliquot sample of 1.5 mL was taken from the just-prepared or just-filtered suspension, put into a disposable cuvette, and immediately placed in the DLS measurement cell. The equilibration time to reach a sample temperature of 25 °C was set to 5 min. The cuvette position was set manually and kept constant for the study. Each sample was measured ten times, and the results were averaged arithmetically. This count rate is multiplied with an attenuation factor to obtain values suitable for comparison. Measurements of the Nanosphere^®^ and silica Levasil^®^ suspensions were analyzed in “multiple narrow” mode, and the other materials were analyzed in “general purpose” mode. In the case of water, 0.8872 mPas was set for viscosity, and a refractive index of 1.33 ± i·0 was used. The refractive index used for the Nanosphere^®^ suspensions was 1.59 ± i·0 according to the documentation sheet. The refractive index used for silica Levasil^®^ was 1.46 ± i·0. The refractive index was 1.64 ± i·0 for both grades of BaSO_4_ (IRMM-381 and IRMM-387), with respect to the wavelength (633 nm) [49]. For the alumosilicate-coated titania (IRMM-388), the refractive index was estimated to 2.77 ± i·0 (without absorption) according to the literature, which means that the contribution of the coating to the particles’ scattering intensity was considered negligible [50]. The refractive index used for kaolin was 1.56 ± i·0 according to the material data sheet. 

Measurements were conducted with the Malvern High Performance Particle Sizer (HPPS) (UK). The HPPS operates with a He–Ne-gas laser (vertically polarized beam, 632.8 nm) and detects scattering light in back scatting mode (173°). The intensity of the illuminating laser beam (“attenuation level” in Malvern terms) was set manually via software to conduct a filtration measurement series with the same laser beam intensity if possible. This laser beam attenuation is realized with an aperture. The measured intensities are captured as detected photons per second and are called count rate. DLS measurements were performed by DLS before and after filtration. An aliquot sample of 1.5 mL was taken from the just-prepared or just-filtered suspension, put into a disposable cuvette, and immediately placed in the DLS measurement cell. The equilibration time to reach a sample temperature of 25 °C was set to 5 min. The cuvette position was set manually and kept constant for the study. Each sample was measured ten times, and the results were averaged arithmetically. This count rate is multiplied with an attenuation factor to obtain values suitable for comparison. Measurements of the Nanosphere^®^ and silica Levasil^®^ suspensions were analyzed in “multiple narrow” mode, and the other materials were analyzed in “general purpose” mode. In the case of water, 0.8872 mPas was set for viscosity, and a refractive index of 1.33 ± i·0 was used. The refractive index used for the Nanosphere^®^ suspensions was 1.59 ± i·0 according to the documentation sheet. The refractive index used for silica Levasil^®^ was 1.46 ± i·0. The refractive index was 1.64 ± i·0 for both grades of BaSO_4_ (IRMM-381 and IRMM-387), with respect to the wavelength (633 nm) [49]. For the alumosilicate-coated titania (IRMM-388), the refractive index was estimated to 2.77 ± i·0 (without absorption) according to the literature, which means that the contribution of the coating to the particles’ scattering intensity was considered negligible [50]. The refractive index used for kaolin was 1.56 ± i·0 according to the material data sheet. 

### 2.7. Calculation Procedures

Grade efficiency curves *T(x)* describe the pore size depended retention properties of filters. *T(x)* is determined by filtration monodisperse RM suspensions and comparisons of count rates, i.e., intensities before and after filtration. The count rate is directly proportional to the quantity of colloidal particles at a certain size. Therefore, the count rate is a suitable measure to estimate the retained quantity of particles and to discuss the retention properties. The quantity of coarse material *g_CR_* was calculated for each membrane type separately [51]. Equation (1) expresses how *g_CR_* is calculated (*n_F_* is the filtrate count rate, and *n_S_* is the feedstock count rate).

(1) $$g_{CR}=1-\frac{\sum n_{F}}{\sum n_{S}}$$

The databased grade efficiency values *T(x)* were calculated for each pore size by means of the count rate amounts of the feedstock suspension *ΔQ_S_(x)* and related filtrates *ΔQ_F_(x)* according to Equation (2).

(2) $$T\left(x\right)=1-\left(1-g_{CR}\right)\frac{\Delta Q_{F}\left(x\right)}{\Delta Q_{S}\left(x\right)}$$

A modified sigmoid function (Equation (3)) is considered suitable to describe the continuous grade efficiency curves *T_Fit_(x)*.

(3) $$T_{Fit}\left(x\right)=\frac{1-\;a}{1+e^{\left(-\frac{x-b}{c}\right)}}+a$$

Curve fitting was done by means of the solver module in MS Excel® and the least squares method. The variables *a*–*c* were calculated for each pore size separately.

Whereas the grade efficiency curve *T_Fit_(x)* describes the size depended retention properties, the intercept of the infection tangent *I(x)* with the x-axis can describe a reasoned size from which filtration starts to have a significant particle size-dependent impact on filtered suspensions. *I(x)* can be described by the linear Equation (4).

(4) $$I\left(x\right)=\frac{a}{4c}\cdot x+\left(\frac{a}{2}+d-\frac{ab}{4c}\right)$$

In order to estimate the intensity-weighted PSDs *Q_F,int_(x)* of the filtered RTM suspensions, the required values were calculated with the results of Equation (5).

(5) $$\Delta Q_{F,int}\left(x\right)=\frac{1-T\left(x\right)}{1-g_{int}}\Delta Q_{S,int}\left(x\right)$$

The required coarse material amount *g_int_* was calculated for each membrane type, and each material separately in the observed particle size range 0.4−10,000 nm, according to Equation (6).

(6) $$g_{int}=\sum T_{i}\left(x_{i}\right)\Delta Q_{S,int,i}\left(x_{i}\right)$$

## 3. Results and Discussion

### 3.1. Membrane Characterization 

**Figure 1:**
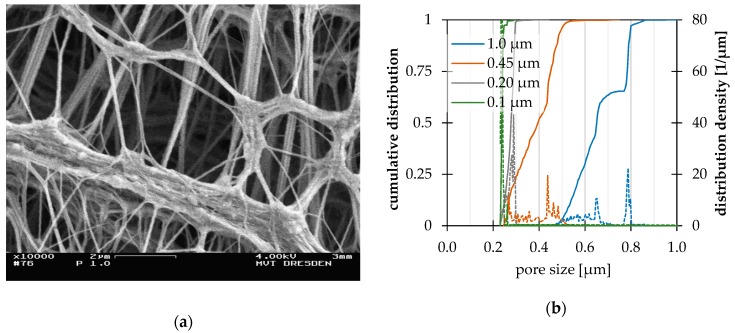
Characterization of OMNIPORE^®^ polytetrafluoroethylene (PTFE) membrane (1.0 µm denoted pore size): (**a**) SEM image after sputtering with platinum in argon atmosphere, magnification 10,000; (**b**) cumulative pore size distributions and distribution densities obtained by the pore size meter PSM 165 by TOPAS GmbH.

The SEM image given in Figure 1 is representative for the membranes in this study and shows fibrous meshes with unregularly formed structures and cavities with various volumes. The observed gap sizes are significantly larger than the denoted value. A capillary pattern, where equal cylindrical and straight pores are assumed, cannot be used to describe to the retention properties. The pores have unequal sizes and might be statistically distributed. The weak pore size uniformity indicates that retention cut offs cannot be absolute. 

Figure 1b illustrates the pore size distribution measurement results of triplicate measurements. The pore size distribution of 1.0 µm and 0.45 µm membranes could be determined reliably, whereas the measurements of 0.2 µm and 0.1 µm membranes hit the lower limit of the instrument (approximately 0.3 µm) and are shown for information purposes only. All membranes show distributions of pore sizes and confirm the SEM image observations. The 1.0 µm membrane shows an absolute size of approximately 0.9 µm, which is below the labelled pore size. The bubble points could be determined for all four membrane types. An overview on the pore size measurement results is given in Table 3 together with the results of filtration studies with polystyrene suspensions.

### 3.2. Blank Samples

The sonicated blank samples and non-treated blank samples were compared according to their count rates and DLS results obtained by the cumulants method. The results are summarized in Table 2. The count rate of non-treated blank samples was always below 60 kcps, which proves that the solution was almost free of particles. A polydispersity index larger than 0.5 indicates that the measured particle size results cannot be considered certain. The contamination after the 1000 nm filtration was very low but led to a stable scattering signal with derived count rates of about 30 kcps. As expected, there is no evidence for contamination during filtration with the 1.0 µm membranes. The filtration was continued with 450 nm pore sizes. DLS measurements of the filtrates show that the additional filtration step leads to slightly increasing count rates, but there is no certain evidence for a systematic and quantifiable contamination during the filtration steps. 

The DLS results of ultrasonic-treated samples show widely spread cumulants diameters that are in the micrometre size range. The high count rate proves that there were many particles generated during the deagglomeration procedure with probe sonication. The polydispersity index (PI) is more than 0.7, which indicates that the measured particle size results cannot be considered certain. After filtration, a significantly decreased count rate is observed. This result indicates that many of the particles generated during sonication are in the size range above 1 µm and, furthermore, that there is a huge contamination in the submicrometre range and probably in the size range of nanoparticles with an unknown amount. This result confirms the already reported size of particulate contamination by ultrasonic dispersion [23]. Contamination in the range of nanometers yields the risk of providing a false positive classification of a material as a nanomaterial. The evaluation of DLS results takes into account that DLS techniques cannot distinguish between the contamination and the sample. Thus, suspensions with count rates close to the blank values given in Table 2 are interpreted as not meaningful for the sample characterization.

### 3.3. Suspension Samples with Reference Materials

This study investigated the membranes retention properties depending on the particle diameter. Nanosphere^®^ polystyrene suspensions with spherical particles in the diameter range 40–900 nm were filtered through OMNIPORE^®^ membranes, and the filtrate was measured by DLS. The filtrate of 900 nm suspensions showed similar count rates to blank samples for all pore sizes, which are interpreted as impurities. In the case of polystyrene (PS) 495, it is remarkable that the cumulants diameter of the filtrate after 0.45 µm filtration was higher than before. The cumulants diameter of the PS 404 suspension was almost constant after filtration through the 1.0 µm and 0.45 µm filters, and the PI was marginally improved. The cumulants diameter of the PS 350 suspension decreased a little after filtration, whereas the PI was nearly constant. These values indicate that a few PS 350 particles with diameters larger than 0.2 µm passed through the filter. The suspensions of PS 40–PS 202 were filtered through 1.0 µm, 0.45 µm, 0.2 µm, and 0.1 µm filters. The cumulants diameters and the PI of these suspensions did not change remarkably.

**Figure 2:**
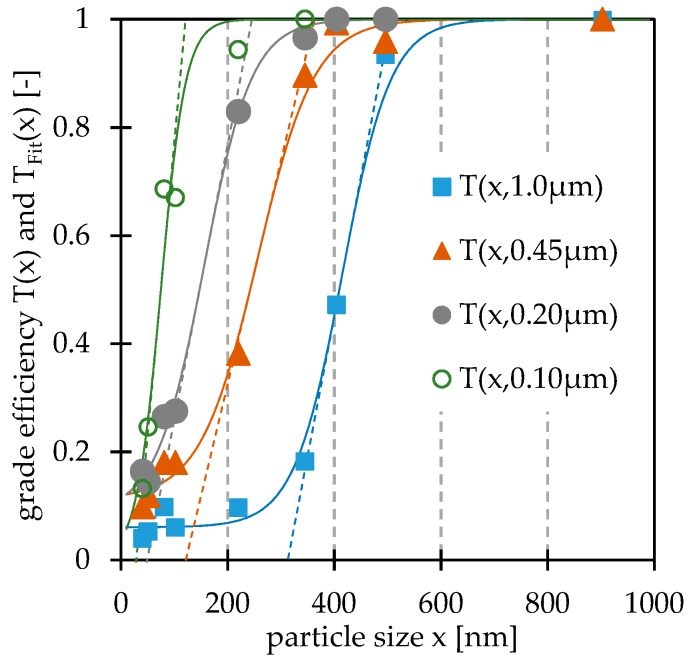
Results of polystyrene suspension filtration studies: Grade efficiencies *T(x)* (data and fitted curves) and related inflection tangents of OMNIPORE^®^ membranes.

Figure 2 shows the grade efficiencies *T(x)* with related inflection tangents obtained with polystyrene suspensions. The values below 10% relative intensity are determined with another laser intensity than the instrument used before in this measurement series. Therefore, values close to x-axis need to be interpreted as rough estimations.

The measured grade efficiency values could be fitted to a modified sigmoid function (Equation (2)). It can be observed that membrane filters always retain particles even if the average pore size is much larger than the particle size. This study proves that filtration always leads to retained particles by diffusion. A summary of related statistical values is given in Table 3. The median grade efficiencies are about half of the median pore sizes obtained by flow measurements. The slope maxima do not vary a lot (0.005–0.011 nm^−1^). In case of the characterization of the 0.1 µm membranethis can also be influenced by the low amount of data points.

The point of intersection of the inflection tangent and the x-axis can describe a borderline diameter and, thus, the transition from non-size selective to size selective retention properties for pore size. This borderline diameter is about a third of the labelled pore size. Concerning the approach of removing unwanted coarse particles only, this can be a safety factor.

The filtration with the 1.0 µm pore size led to significant impacts on the particle quantity of PS 900, PS 495, and PS 404 suspensions. The quantity of PS 350 particles decreased by 18.2%, which is still a lot, but there was no change of cumulants diameter and PI. Therefore, this filtration step might be accepted in this case. This perception verifies a size safety factor of approximately three. In the case of the 0.45 µm pore size filtration, the quantity of PS 202 decreased by 38.2%, but the cumulants diameter and PI did not change noticeably. Because of this unremarkable change of the average size, a size safety factor of 2.5 might be acceptable if there is no need to keep the particle quantity as high as possible. In all other cases, a filtration at the 0.45 µm pore size cannot be recommended for PS 202, but it can be recommended for PS 100 and smaller particle sizes. In the case of the 0.20 µm filtration, the quantities of PS 100 and PS 80 decreased by approximately 27%, but the quantities of PS 50 and PS 40 decreased only approximately 15%. These observations prove that a size safety factor of three is suitable to keep the particle quantity as high as possible. 

### 3.4. Effect of Particle Concentration

The grade efficiency functions were determined for dilute suspensions (100 ppm). The filtration of higher concentrated suspensions yields the risk of cake filtration because of the fast blocking of pores. The consequences were low permeability and higher filter resistance than before. For the purpose of sample preparation, separation should be achieved by sieving and depth filtration. Both mechanisms are connected to relatively low particle concentrations. This study investigates the grade efficiency in the dependency of the solid content to determine the applicability range regarding particle concentration. Monomodal silica suspensions (Levasil^®^) in the solid content range 350–3500 ppmw were filtered, and the filtrate was measured. The results are summarized in Table 4.

The initial solid content had a little but significant effect on the retention properties. In the case of the lowest concentrated sample, the initial cumulants diameter decreased from 105 ± 2 nm to 98 ± 1 nm, whereas, in the case of the highest concentrated sample, the cumulants diameter decreased but not significantly. The PI did not change remarkably, and no tendency was noticeable. This relation shows that high concentrations might be more suitable for filtration than diluted suspensions. The ratios (350 ppm/1000 ppm/3500 ppm) of the mean count rates changed only a little from 1:2.9:10.8 to 1:2.6:9.4 (1.0 µm), 1:2.6:9.3 (0.45 µm), 1:2.4:9.4 (0.2 µm), and 1:2.4:9.9 (0.1 µm). In the case of 1.0 µm filtration, a slightly higher count rate (+3.4%) was measured for the 350 ppm suspension, which is in the uncertainty range. The results indicate that diffusion loss is the most important effect in this solid content range. For this ratio of pore size to particle size, the impact of blocking is negligible. 

### 3.5. Representative Materials

**Figure 3:**
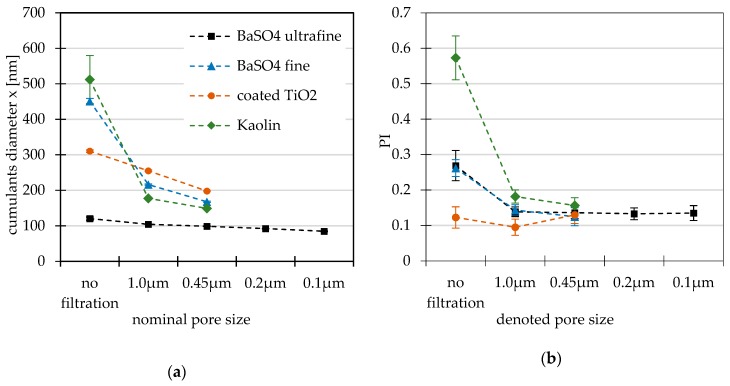
Averaged results of DLS measurements of RTMs of feedstock suspension and after filtration with PTFE membranes: (**a**) Mean cumulants diameter; (**b**) mean polydipersity index (PI).

This study investigates the filtration of four RTMs in the nano- and non-nano size range. The suspensions were prepared and measured in triplicates, i.e., three independently prepared samples, and each of them was filtrated and measured ten times by DLS. Figure 3 illustrates the DLS results of the feedstock suspension and filtrate obtained by means of cumulants method. 

The mean cumulants diameters of the feedstock suspensions of the coated titania and both grades of BaSO_4_ indicated a good reproducibility of the sample preparation procedure. Only in case of kaolin did the cumulants diameter vary a lot (511.8 ± 36.8 nm), and the PI was very high (0.57 ± 0.01). Filtration led to significantly decreased mean cumulants diameters of all non-nano RTMs. The PI was considerably improved for kaolin and both grades of BaSO_4_, but it was not remarkable for coated titanium oxide, which was already acceptable. In the case of the BaSO_4_ fine suspension, the count rate decreased to 6055 ± 2581 kcps after the filtration procedure. This high impact on the count rate indicates that a high quantity of particles was retained. Continued filtration (0.45 µm) led to continuously decreased mean cumulants diameters for all RTMs. The PI did not change significantly anymore. 

**Figure 4:**
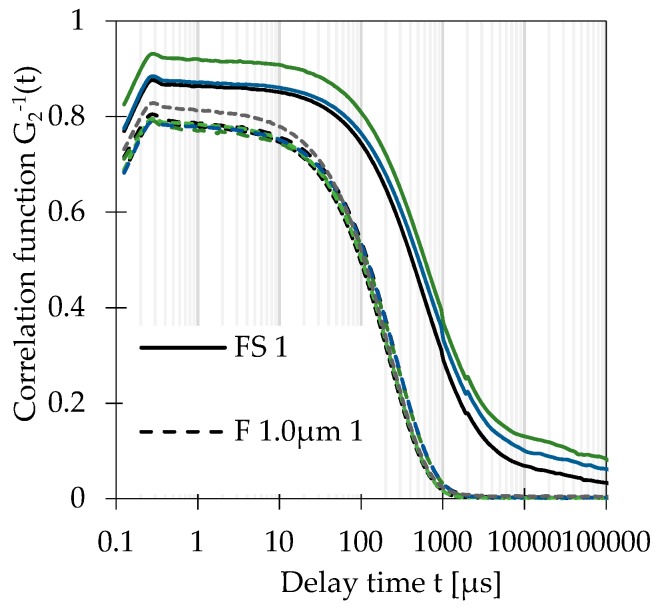
DLS correlation functions *g_2_^−1^(t)* of all replicates of kaolin suspensions: Feedstock suspensions (FS) and filtrates (F) obtained with OMNIPORE^®^ PTFE membranes (1.0 µm and 0.45 µm pore sizes).

Figure 4 shows that correlation functions *g_2_^−1^* could be improved for kaolin, i.e., a textbook-like mono-exponential decay was achieved, while the correlation functions of original samples were shifted along the ordinate because of the presence of micrometre sized particles, which caused an additional relaxation mode at very large decay times. [17]

**Figure 5:**
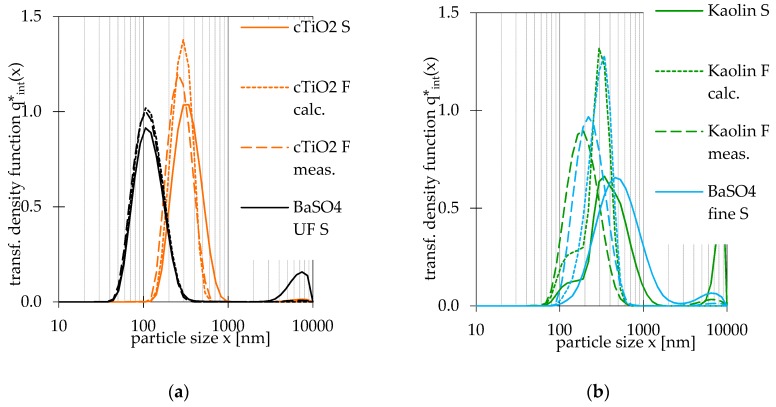
Comparison of intensity-weighted PSDs of feedstock (S) and 1.0 µm pore size filtrate (F): (**a**) Coated TiO_2_ and BaSO_4_ ultrafine (UF); (**b**) kaolin and BaSO_4_ fine.

In order to investigate the predictability of the filtrate’s PSD and the enhancement of sensitivity for fine particle, the filtrate’s PSD were calculated according to Equation (5). The amount of large particles was calculated according to Equation (6). Measured and calculated filtrate’s PSDs at 1.0 µm pore size are compared to each other in Figure 5. 

In the case of BaSO_4_ UF, the calculated and measured PSD are similar to each other, which proves that the grade efficiency is basically valid in the size range 40–400 nm. In the case of submicrometre RTMs, all PSDs were cut at approximately 800 nm. The minimum and maximum of calculated and measured filtrate’s PSDs are nearly equal for each material, but the measured peak diameter is always smaller than calculated one. Hence, the sensitivity could be improved for finer particles and thus the measured higher quantities of small particles. In case of kaolin, an almost bimodal PSD was calculated, but a monomodal PSD was measured, which is probably caused by smoothing due to instruments algorithms. All these effects lead to smaller average diameters than calculated. The conversion to other types of quantity yields the risk of falsification of PSDs because of implications that happen due to assumptions of Mie’s model, as well as fitting and smoothing by instrument algorithms. Nonetheless, the impact of filtration on the number-weighted and volume-weighted PSDs varies. A median diameter shift to smaller values can be observed for types of quantity for all RTMs. This shifts of x_50,0_ are smaller those of x_50,3_ because coarse particles have a significant impact on the volume-weighted PSD but a low impact on the number-weighted PSD. In the case of BaSO_4_ UF, no shift was noted for x_50,0_. Filtration with a 1.0 µm and even 0.45 µm pore size can be recommended for a material in the nano-sized range if this is required for any reason.

**Figure 6:**
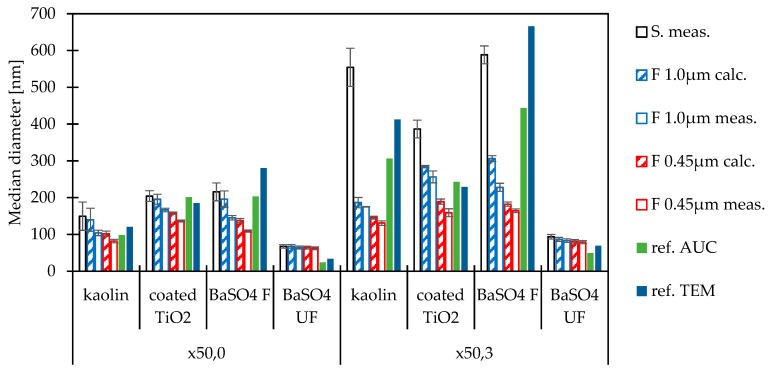
Comparison of measured and calculated median diameters of filtrates with reference diameters from the literature: Volume-weighted median diameter x_50,3_ and number-weighted median particle diameter x_50,0_.

In Figure 6, the median diameters x_50,3_ and x_50,0_ (DLS) are compared to results obtained by analytical ultracentrifuge (AUC) in a refractive index measurement mode and transmission electron microscopy (TEM) [16]. It should be noticed that there is no assured agreement between the diameters measured by AUC and TEM, but the agreement between these size determination methods is considered sufficient according to the state of art.

In the case of kaolin, the filtrate’s particle diameter x_50,0_ (DLS 104.2 ± 7.5 nm) shows a satisfying agreement to the diameter x_50,0_ obtained by AUC (98 nm) and a similarity to the TEM result (120.6 nm). However, the maximum diameter of the main peak is approximately 1718 nm, which is much higher than the membranes pore size. The materials size ratio (min/max) according to DLS is approximately 29 (nm/nm). Filtration retains too many particles to determine a reliable volume-based diameter x_50,3_. Hence, a filtration with 1.0 µm pore size can be recommended for a material in the expected average size range of at 100 nm for the determination of x_50,0_ only. Filtration is not considered suitable for materials with broad PSDs with a diameter maximum in the micrometre range. In the case of coated titanium dioxide, the diameter x_50,0_ (DLS) of the unfiltered suspension (204.2 ± 14.4 nm) is a little higher than the diameters determined by AUC (201 nm) and TEM (185 nm). The filtrate’s diameter x_50,0_ (166.5 ± 4.9 nm) is not closer to the diameters obtained by AUC and TEM. In contrast, filtration leads to a much better agreement of the x_50,3_ (256.3 ± 16.1) to the results by AUC (243 nm) and TEM (228.8 nm). The materials’ size ratio (min/max) according to DLS is approximately 16 (nm/nm). Hence, filtration before DLS measurement can probably be recommended for a submicrometre material with narrowly distributed PSDs in this size range. In the case of BaSO_4_ fine, x_50,0_ of the unfiltered suspension (215.7 ± 24.3 nm) is close to the values obtained by AUC (203 nm) and SEM (212 nm). Filtration and measurement by DLS lead to much smaller diameters, and, thus, filtration cannot be considered suitable for this material. The maximum of the main peak is by approximately 2669 nm in the intensity-weighted PSD, and the size ratio is approximately 34 (nm/nm). Filtration retains too much particles to determine a diameter.

The study results are consistent with the general belief in the nanomaterial analysis community that a single particle measurement method cannot cover the required size range from a few nanometers to well beyond one micrometer to determine the “real” size distribution of a particulate substance for all kinds of materials [52]. If the existence of micrometre particles is expected, the application of a laser diffraction or static light scattering technique (LD/SLS) might be recommended to get a first impression of the particle size distribution width and to assay the suspension on particles, aggregates, and agglomerates in the upper submicrometre and micrometre range. The use of LD measurements prior to DLS analysis might be implemented in an operating procedure as a suitable routine to confirm or reject the assumption of large particles, to verify whether filtration is needed, and to estimate the impact of filtration on the size distribution.

The findings could be generalized for membrane filters comparable to the hydrophilic PTFE membranes used in this study and similar polymers with a comparable mesh structure. The use of other membrane materials, e.g., mixed esters of cellulose (MCE), polyvinylidene fluoride (PVDF), or polyethersulfone (PES), could lead to other physico-chemical interactions between the particles and the filter. Only membrane materials with a negative surface charge should be used for suspensions stabilized with anionic surfactants. The authors recommend to conduct experimental studies on the retention properties if another material than PTFE is used.

## 4. Conclusions

The effects of microfiltration have been studied in order to improve DLS for particle size determination. Filtration was carried out with particulate RMs and RTMs in the nano- and submicrometre range. The differential pressure and filter area were kept constant in this study. Polystyrene standards (40–900 nm diameter) were used to investigate the retention properties of PTFE filters with denoted pore sizes in the range 1.0–0.1 µm. The results were fitted to a sigmoid function. A size safety factor of at least three for monodisperse materials was determined to keep separation as low as possible. However, filtration always retains particles by diffusion and, therefore, leads to a decrease of quantity. Concentration implications were investigated with a monomodal silica suspension in the solid content range 350–3500 ppm. The study demonstrated that concentration effects play a minor role in this application range. 

RTMs with broad PSDs were used to investigate the filtration procedure. There are potentials of improving correlation functions and thus increasing the reliability of the DLS method. Filtration leads to modifications of the granulometric state and thus shifts of median diameters. In the case of submicrometre materials, the volume-weighted median shifts to a large extent because of the retention of contaminations and large sample particles. Regarding the number-weighted median diameter, this shift is less extensive.

The comparison of DLS results with those obtained by AUC and TEM showed that filtration before DLS measurement can improve the agreement of median diameters. In case of materials with diameters close to or below 100 nm, filtration with membranes (1.0 µm pore size) can be recommended. Materials with submicrometre particles are potentially suitable. In the case of narrowly distributed PSDs, the determination of median diameters x_50,0_ and x_50,3_ could be improved. In the case of particulate materials with micrometre particles, the determination of the median diameters x_50,0_ by DLS can be improved under certain circumstances only. Experiments showed that a suitability is given if the material’s PSD is not too wide. However, further studies with other membrane materials are needed to generalize the findings in order to establish a possible routine for sample preparation for DLS measurements.

## Figures and Tables

**Table 1:** Representative test materials (RTMs) used in this study. Reference diameters obtained by analytical ultracentrifuge (AUC) and transmission electron microscopy (TEM) were taken from the literature.

Material	Supplier	Reference diameter by AUC [nm]		Reference diameter by TEM [nm]	
		x_50,0_	x_50,3_	x_50,0_	x_50,3_
BaSO_4_ ultrafine grade (UF) (IRMM-387)	JRC	24	49.3	33.4	69.3
Levasil^®^ 50/50%	H.C. Starck	-	-	-	-
Kaolin (IRMM-385)	JRC	98	306	120.6	412.2
Coated titania (IRMM-388)	JRC	201	243	185.0	228.8
BaSO_4_ fine grade (F) (IRMM-381)	JRC	203	444	280.5	665.6

**Table 2:** Comparison of dynamic light scattering techniques (DLS) results between samples treated with ultrasonic devices and non-treated samples.

Membrane Pore Size [µm]	Non-Treated Blank Samples			Sonicated Samples				
	Count Rate [kcps]	x_cumulants_ [nm]	PI	Count Rate [kcps]	x_cumulants_ [nm]	PI	x_50,int_ [nm]	x_50,0_ [nm]
no filtration	32.3 ± 21.3	387 ± 351	0.77 ± 0.15	9497 ± 392	1352 ± 164	0.88 ± 0.07	446.6 ± 33.7	102.3 ± 12.3
1.0	30.4 ± 3.8	695 ± 308	0.77 ± 0.14	1362 ± 179	152.2 ± 1.6	0.29 ± 0.02	160.5 ± 1.8	66.9 ± 2.3
0.45	124 ± 68.3	342 ± 89	0.63 ± 0.16	820.8 ± 88	127.1 ± 4.3	0.25 ± 0.03	136.7 ± 1.9	52.2 ± 2.6

**Table 3:** Results of membrane characterization studies obtained with pore size meter PSM 165 and polystyrene filtration studies (median grade efficiency and the intercept of the inflection tangent with the x-axis). Hardly reliable values that hit the limit of quantification are represented in italics.

Denoted Pore Size [µm]	Bubble Point (Pressure) [µm, (mbar)]	Median Pore Size [µm]	Modal Pore Size = [µm]	Grade Efficiency Median µm]	Inflection Tangent at I(x) = 0 [µm]
1.0	1.03 (446)	0.74	0.81	0.409	0.313
0.45	0.76 (606)	0.39	0.44	0.242	0.122
0.20	0.44 (1029)	*0.28*	*0.29*	0.147	0.049
0.10	0.30 (1535)	*0.24*	*0.24*	0.074	0.029

**Table 4:** DLS results of silica suspensions (Levasil^®^) with different solid contents filtered with OMNIPORE^®^ membranes. All measurements were carried out with same laser intensity.

Membrane Pore Size [µm]	350 ppm			1000 ppm			3500 ppm		
	Count Rate [kcps]	x_cumulants_ [nm]	PI	Count Rate [kcps]	x_cumulants_ [nm]	PI	Count Rate [kcps]	x_cumulants_ [nm]	PI
no filtration	15806 ± 97	105 ± 2	0.092 ± 0.034	45399 ± 319	107 ± 1	0.056 ± 0.019	171361 ± 2056	106 ± 1	0.073 ± 0.015
1.0	16343 ± 133	104 ± 2	0.087 ± 0.029	43131 ± 728	105 ± 1	0.06 ± 0.017	154329 ± 2724	106 ± 0	0.077 ± 0.017
0.45	14898 ± 142	102 ± 1	0.106 ± 0.013	38985 ± 401	104 ± 1	0.073 ± 0.020	138787 ± 1047	105 ± 1	0.079 ± 0.020
0.20	13549 ± 59	101 ± 2	0.087 ± 0.047	32958 ± 223	102 ± 1	0.072 ± 0.012	127992 ± 573	105 ± 1	0.066 ± 0.016
0.10	11629 ± 69	98.4 ± 1	0.110 ± 0.027	27362 ± 279	101 ± 0	0.060 ± 0.013	116059 ± 750	104 ± 1	0.060 ± 0.018
